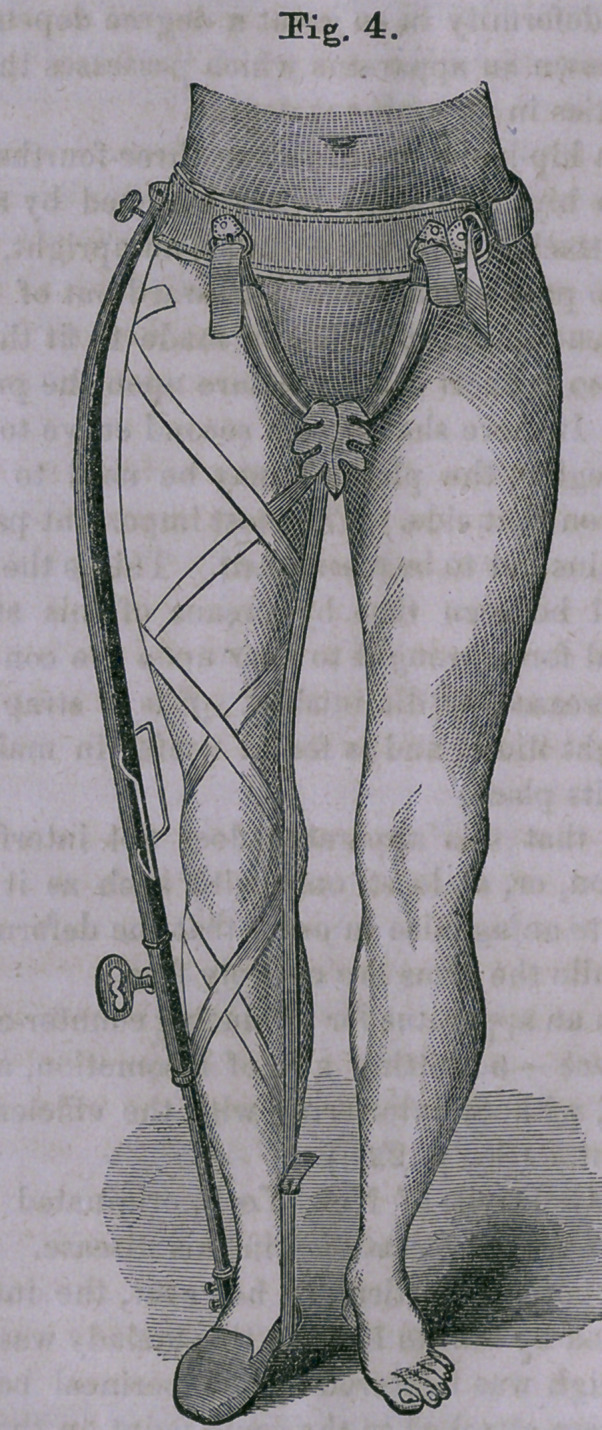# The Recognition of American Surgery Abroad

**Published:** 1868-04-01

**Authors:** F. O. Earle

**Affiliations:** Chicago, Ill.


### CHICAGO MEDICAL JOURNAL.

*Vol. XXV.—APRIL* 1, 1868.—*No.* 7.

### THE RECOGNITION OF AMERICAN SURGERY ABROAD.

*With a Brief Consideration of the Indications for Treatment in Certain Deformities, and a Description of Orthopædic Apparatus Exhibited at the Paris Exposition.*

BY F. O. EARLE, M.D., CHICAGO, ILL.

THE decided preference given to American improvements in surgery at the late Paris Exposition, is a most gratifying proof of the progress of medical science in this country.

This official recognition of the superior excellence of these productions of American genius over corresponding European contributions, though obtained by individuals, must, nevertheless, be looked upon as an honor to the medical profession in America, as a whole, and is an event which should awaken a feeling of pride in the heart of every member of it; for we have long been accustomed to look abroad for all scientific excellence, and especially in all things pertaining to medicine and surgery; and this triumph of American over European ideas is the more satisfactory when we remember that it was gained on European ground, and that the judges in this great international contest were Europeans.

In the important departments of the healing art, more especially, were American physicians the successful competitors; viz., in military surgery, and in orthopædic surgery.

The American Sanitary collection of Dr. Evans, comprising every thing which can contribute to the physical welfare of the soldier, whether in camp or on the march, on the field or in the hospital, elicited unqualified admiration and praise; and all these improvements have been unanimously adopted by the European powers.

Space will hardly permit here an examination of the articles included in this collection; but the "Howard Ambulance" was, on account of its very superior merits, looked upon with special favor, and having been awarded a silver medal, deserves particular mention. The whole collection received the highest prize of a gold medal.

In a communication which appeared in a late number of the Journal, (Oct., 1867,) I observed that the most useful discoveries and important practical suggestions regarding the management of deformities, had been made by American physicians. The names of Drs. H. G. Davis, L. A. Sayre, and C. F. Taylor, all of New York, are as "familiar as household words" in the history of the progress of this important department of surgery in this country, and each of them have furnished valuable additions to its literature.

By referring to the official report of the Imperial Commission, appointed to examine surgical instruments and apparatus, a partial translation of which is given below, it will be seen that to Dr. Taylor is given the honor of having exhibited the *only* improvements in apparatus for the treatment of deformities.

The report reads as follows:

"In orthopædy (that branch of surgery relating to the treatment of deformities), there is nothing new in the Exposition, except the collection of apparatus of Dr. C. F. Taylor, of New York. Of these apparatuses, some are intended to correct vertebral deviations consequent upon Pott's disease, or angular curvature of the spine, and others for lateral curvature; others, too, are for the cure of muscular paralysis of childhood, by means of localized movements. Dr. Taylor has on exhibition models of extremely ingenious apparatus, which promote the development of certain muscles or groups of muscles by means of local exercise.

"The spinal apparatus of Dr. Taylor is remarkable, and differs entirely from analogous apparatus in the Exposition."

At a meeting of the Academy of Medicine, of Paris, held subsequent to the close of the Exposition, the attention of its members, was called to the appliances by M. Bouvier, "one of the most competent French surgeons," whose observations on their merits and the advantages to be derived by the patient from their employment, were embodied in the report of the commission.

Having employed most of the apparatuses in my own practice, I can speak in the highest terms of their genuine practical utility; and presuming that a description of some of them may not prove uninteresting to the readers of the JOURNAL, the limits of a single article not sufficing for a representation of the whole, I have selected those for spinal curvatures and morbus coxarius, as possessing unusual interest for the general practitioner, whose attention is not unfrequently called to those maladies of most common occurrence in the practice of the orthopædic surgeon.

Before describing the several instruments, it will be necessary to consider, very briefly, the pathology of the diseases to which they are applicable, and the indications for their employment.

In this disease, "Pathological Anatomy shows us an intervertebral cartilage in a condition of congestion or softening, yielding to the pressure from above; the body of an inflamed or ulcerated vertebra crumbling anteriorly under the superimposed weight of the head, trunk, and upper extremities, and thus producing a posterior projection of the spinal column."*

The indications in this disease, I conceive to be:

* Contributions to the Pathology, Diagnosis and Treatment of Angular Curvature of the Spine. By BENJ. LEE, M.D. Philadelphia, 1867. P. 62.

To afford to the vertebral column effectual relief from pressure at the diseased point.

To secure the bodies of the affected vertebra against the irritation arising from motion and concussion, while the patient maintains the erect attitude.

To assist the vertebral column to regain the true spinal tone; and

To rectify all constitutional disturbances.

As these constitutional disturbances are, in a very large majority of cases, directly dependent upon the diseased condition of the spine, and the deformity consequent thereupon, it is natural to infer that they will be relieved if the indications previously mentioned are fully answered; and experience proves this inference to be correct.

It becomes, important, then, to inquire how these indications may be most satisfactorily fulfilled.

Fig. 1.

Fig. 2.

Figs. 1 and 2 represent a front and back view of an apparatus for the treatment of angular curvature of the spine.

In my own practice, I have never found any means so effectual as the apparatus shown in the above cut, which consists of a broad, well-padded band, which embraces the trunk low down. From this hip-band, two steel uprights pass up the back on either side the spine, and these are attached at the top to a double-T-shaped crosspiece. Opposite the point of disease are two broad plates, which are attached to the uprights by stop-hinges; and outside of these, above and below, are little screws, which pass through the upright and rest upon the pad-plate.

A pair of shoulder-straps, made of firm soft webbing, and padded; a broad apron, nearly covering the anterior portion of the trunk; and a pair of pads, which are fastened to the instrument opposite the seat of disease — the point of which a fulcrum is made — complete the apparatus.

The ends of the cross-piece at the top, to which the shoulder-straps are fastened, are spread far apart, the upper one resting just below the slope of the shoulder, and the lower reaching a point on a level with the axilla.

The object of this arrangement is that the straps may pass directly forward and around the arms, thus preventing the loss of any force by diagonal action, and also entirely obviating any painful and injurious ligaturing of the arms.

The chief peculiarity of this instrument is the arrangement of hinges by which the pad-plates are attached to the uprights, and the little screws passing through the latter above and below the hinges, and working against the pad-plate.

By turning in the screws, the hinges are opened, and the amount of sustaining force increased to any desirable extent.

The hinges, by their position, absolutely prevent motion forward, but are "free to bend backward," thereby making special provision for the exercise and development of the spinal muscles. These organs are thus made accessory to the apparatus in straightening the curved spine; for the "spinal muscles, by alternate action and rest, actually alternate with the instrument in sustaining the weight of the body and overcoming the curvature." *

* The Mechanical Treatment of Angular Curvatures. By Charles Fayette Taylor, M.D. New York, 1864. P. 26.

It will be seen that the action of this apparatus is directly backward at the hips and shoulders, and directly forward at the point of disease; and that, therefore, no force is lost, but it is all exerted in a direction tending to straighten the spine.

The limits of this article will not allow — even were science to be promoted thereby — a full consideration of the theories, more or less plausible, which have been advanced, from time to time, regarding the pathology of lateral curvature of the spine.* Incipient cases, which alone could disclose the true pathological condition of the textures involved, can very rarely be obtained for examination; and advanced cases, when opportunity is afforded for examining them, reveal results merely, without yielding any evidence respecting causes.

When the truth is thus involved in obscurity, we can only rely upon such facts and deductions as may be established by extended and multiplied observation; and these seem to indicate that in these cases the spinal column is mechanically, not pathologically, affected — that none of the tissues of the spine itself are actually diseased.

The vertebral column is exceedingly flexible; and its muscles failing, by reason of undue weakness, to give it adequate support, it consequently bends; and this tendency to deviation is constantly increased by the superincumbent weight, which, if continued long enough, produces permanent deviation of the column.

The apparent cause, then, of this distortion is weakness of the spinal muscles. But the organs can hardly be considered in a pathological condition merely because they are suffering from a certain degree of debility. There must be some reason for this weakened condition of the muscles, and this will also be found to be the *real* cause of the deformity.

Dr. Taylor, in a paper read before the New York County Medical Society, at its stated meeting, May 7th, 1866, and entitled "The Initial Cause of Lateral Curvature of the

* Reference is not made here to those cases of lateral curvature—comparatively few in number—caused by rachitis, paralysis, reflex spasmodic contraction of the muscles, and unequal length or distortions of the lower extremities.

Spine," observes: "This form of curvature we are now considering, often occurs where there is every evidence of active nutrition and growth." *

We have seen that none of the tissues involved in the organization of the spine itself are diseased; neither does it appear that the muscles whose duty it is to support the spine in its erect position, have suffered any pathological change.

Where, then, shall we look for the cause of this deformity?

In reference to this, the writer above quoted says: "To one source only, then, can be traced the initial cause of lateral curvature—the nervous system. The muscles fail to give support to the column, from deficient innervation." †

This conclusion is amply supported by the facts connected with these cases: to wit, the age at which the curvature generally begins; the immense expenditure of nervous force to which the patient is subjected at this period of most rapid growth and development, when all the energies of the system are required to fit her for the duties and functions of womanhood, but which are too lavishly expended in the cultivation of the mind, and the attainment of the polite accomplishments, thus entirely subordinating the physical to the mental organization, and straining the brain and nervous system to their utmost tension, at a time when the patient has little nervous force to spare.

This view finds additional support in the happy results of treatment in many incipient cases, in which the directions have been simply to remove the patient from school, have her lie down much of the time, carefully avoid all mental labor and excitement, and, in fine, do nothing but grow.

If the foregoing deductions are true, the indications are plain. They are:—

To protect the patient from waste of nervous force.

To overcome the manifest debility of the spinal muscles.

To relieve the lateral portions of the spinal column from pressure.

* N. Y. Med. Record, Vol. 1, No. 7, p. 165. † *Ibid.*

We find our means of fulfilling these indications in entire rest from mental activity, in "localized movements," in mechanical appliances.

The first of these measures must be absolutely insisted upon in all cases. There must be an entire interruption to mental labor, and excitement of all kinds must be avoided.

"Localized movements" can not well be dispensed with in the treatment of this form of curvature, their use being twofold: to strengthen the muscles, and to relieve the deformity.

In incipient cases, the means above indicated will generally be found sufficient for the cure; but in cases more advanced, mechanical support is often necessary.

Fig. 3.

An instrument to be efficient in reducing lateral curvature, must be constructed with the view of exerting lateral pressure, and, when necessary, counter-pressure; care being taken that we do not prevent or hinder necessary muscular exercise, and thereby hasten the progress of the deformity which we are endeavoring to relieve, by increasing the muscular weakness upon which the deformity in so great a degree depends.

In Fig. 3 is shown an apparatus which possesses the above-mentioned qualities in an eminent degree.

It consists of a hip-band, *a*, extending three-fourths the distance around the hips, the ends being attached by a padded leather strap. Attached to the hip-band is an upright, *b*, which is curved so as to pass upward and backward out of the way, and ends in a broad plate, *c*, which is made to fit the side of the trunk, and also to exert some pressure upon the prominent shoulder-blade. If there should be a second curve to the left, in the lumbar region, the plate, *d*, may be used to embrace the floating ribs on that side. The most important part of the instrument remains yet to be mentioned. This is the perineal band, *e*. It will be seen that by means of this strap, the amount of lateral force brought to bear upon the convexity of the curve is increased or diminished. *f* is a strap made to pass over the right ilium, and is found useful in maintaining the hip-band in its place.

It is apparent that this apparatus does not interfere with any bodily motion, or, at least, only with such as it is absolutely necessary to antagonize in order that the deformity may be overcome, while the arms are entirely free.

Fig. 4 exhibits an apparatus for producing counter-extension in hip-joint disease — admitting also of locomotion, and rotation of the limb, without interfering with the efficient action of the instrument. (See p. 228.)

With Dr. H. G. Davis, of New York, originated the idea of *counter-extension with locomotion* in this disease.

In several important particulars, however, the instrument proposed and used by him in healing this malady was far from perfect. The thigh was ligatured by the perineal band, both ends of which were attached to the same point on the shaft of the instrument; and he also employed "elastic extension."

Dr. Taylor observed these faults, and proposed to remedy them by means of a cross-piece and joint at the pelvic end of the apparatus, and by using unyielding force in place of elastic extension.

Fig. 4.

This was a great advance towards perfection, but there was still wanting one element to make the instrument complete.

The sole object of counter-extension is to destroy the tonicity of the flexors, abductors and adductors of the thigh, and thus relieve the pressure upon the surface of the joint.

If this object be perfectly accomplished, the joint will be preserved from shock and pressure, and the patient can have the advantage of fresh air and exercise, as under these circumstances locomotion is not only free from danger, but is eminently advantageous.

Dr. Taylor's first modification of Davis' splint, though a great improvement upon the latter, was still imperfect in this respect: that the adductors, the muscles most influenced by contractions, were not perfectly antagonized; and on account of this inability of the instrument to extend these muscles to a sufficient degree to overcome this tonicity, apparent shortening of the limb would necessarily occur.

This object (perfect antagonism of adductors), so desirable, and, in fact, absolutely essential to a complete recovery in these cases, is fully accomplished by the apparatus shown in the cut.

Here, it will be seen, the cross-piece at the pelvic end of the instrument is converted into a hip-band, which embraces the hips for about three-fourths of their circumference, and is terminated by a leather strap, covered with a removable pad to guard against pressure and abrasion. Attached to the hip-band, in such a manner as to admit of flexion and abduction, is the shaft, which passes down the outer side of the leg, and at the external malleolus is inserted into the foot-piece, which will be described more particularly hereafter.

It will be noticed that in this appliance, two perineal bands are used. The advantage of this arrangement is two-fold. 1st, By tightening the opposite strap, the thigh is very strongly abducted, and thus we act directly on the adductor muscles. 2nd, The danger of abrasion of the skin in the perineum is diminished in a great degree; and if it should become sore, one strap can be loosened, or removed altogether, if necessary, and the action of the instrument kept up temporarily by the other. Abduction will always be rendered more certain, by having a small thumb-screw passing through the upper end of the shaft, and working upon a steel plate on the hip-band. On the shaft of the splint, opposite the knee, is a bent wire, which serves as a point of attachment for a knee-cap, or pad, not represented in the engraving.

The lower end of the shaft is received in the foot-piece, and is held in place by the screws, the upper one clamping a shallow depression roughened at the bottom, and the lower fitting loosely in a deep groove in the end of the shaft. The foot-piece is riveted to the sole of the shoe, and contains two slits, one on each side of the foot, through which passes a piece of webbing, by which the outside adhesive strap is terminated, and which is fastened to the inner strap, terminating in a buckle.

As the action of the instrument must be exerted unremittingly, it is necessary to have two foot-pieces exactly alike, one attached to the shoe for day use, and the other to a slipper to be worn at night.

Extension is made by means of the key which fits a ratchet on the shaft, and after being extended to such a degree as is required to make the patient comfortable, it is fastened by the slide.

After the adhesive straps are applied, as represented in the figure, the limb is bandaged from the ankle to the hip.

In presenting these appliances to the notice of the profession, it has not been my intention to convey the idea that they will, in the exact form here represented, answer for all cases.

Our remedies must always be the embodiment of our ideas of the special requirements of the particular case which is before us. Routinism in the practice of any department of medicine, can not be too severely reprehended.

All the instruments which have been described are capable of unlimited modification, and they must be used discreetly, intelligently, and, above all, *efficiently*, if we expect to gain the best results.

## Figures and Tables

**Fig. 1. f1:**
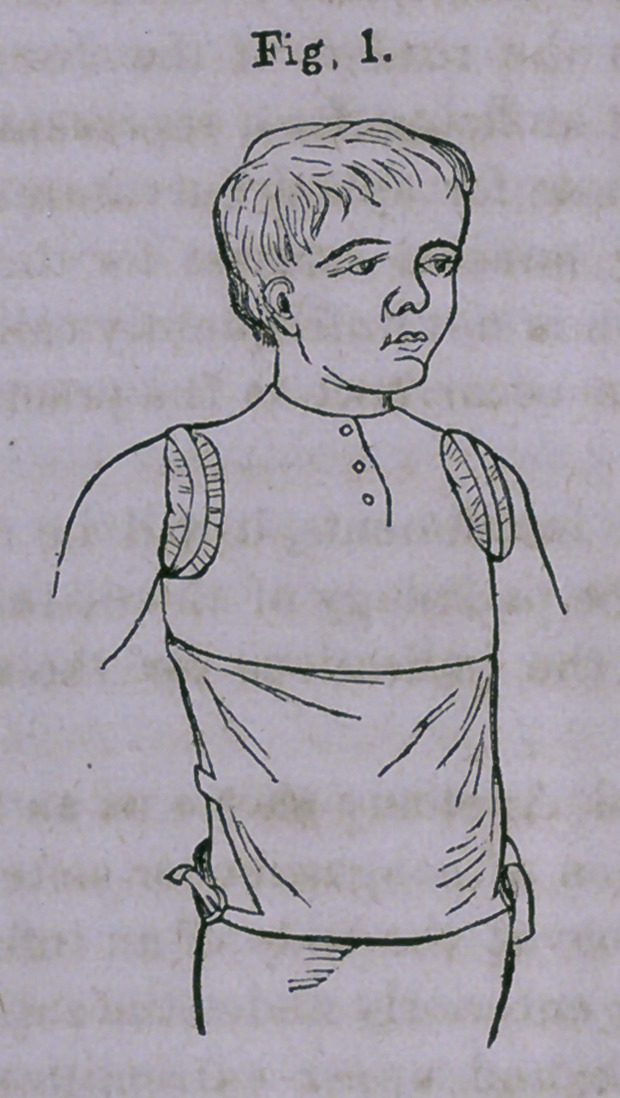


**Fig. 2. f2:**
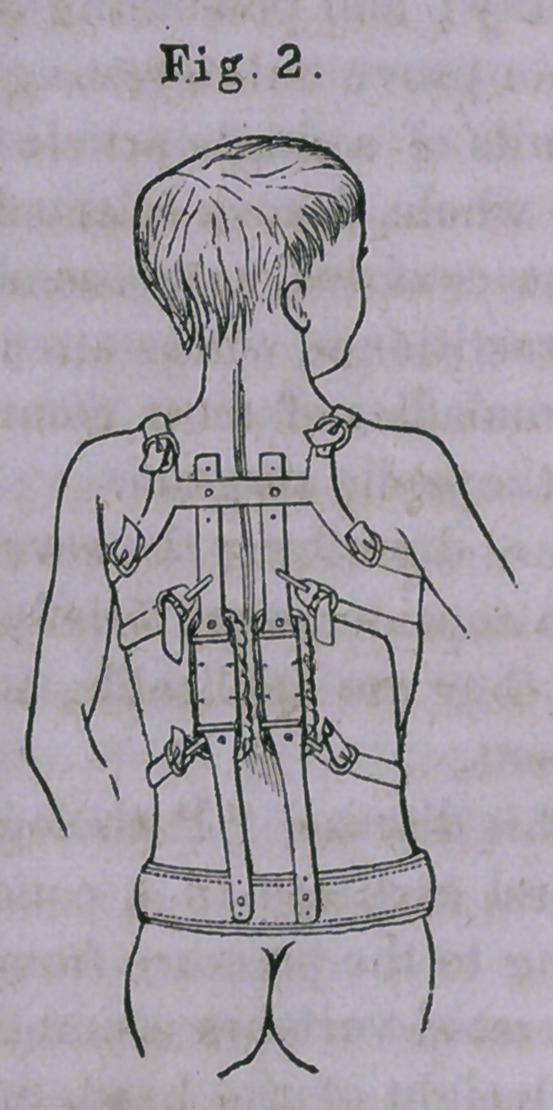


**Fig. 3. f3:**
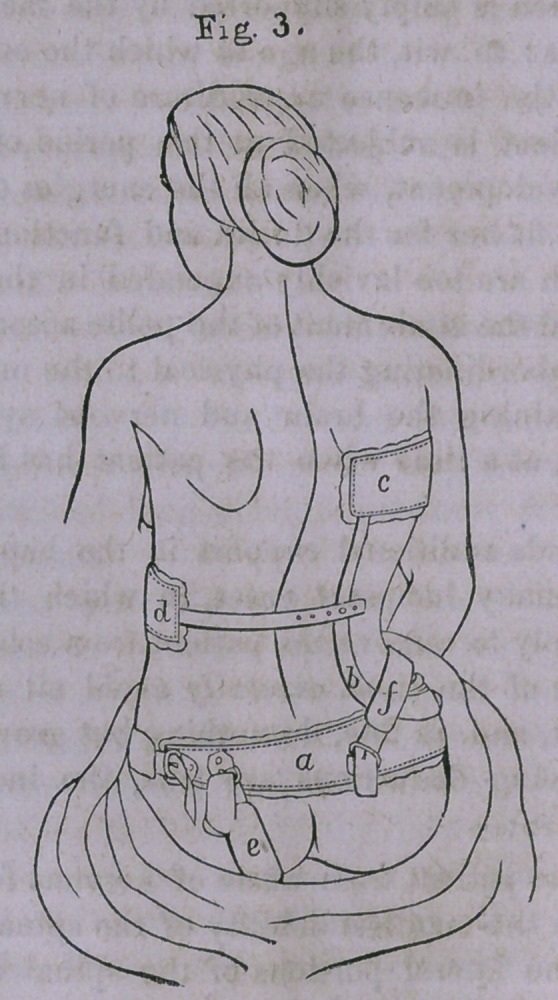


**Fig. 4. f4:**