# Errata

**Published:** 1868-05-01

**Authors:** 


### *Errata.*

(Dr. Earl's Article.) Page 220, first line: for "the important," read "two important."

Page 221, seventh line from bottom: for this disease," read "Pott's disease."

Page 222, seventh line from top: for "tone," read "line."

Page 227, fourth line from bottom: for "healing," read "treating."

